# Wine Yeasts 1.0

**DOI:** 10.3390/microorganisms10010026

**Published:** 2021-12-24

**Authors:** Matthias Sipiczki

**Affiliations:** Department of Genetics and Applied Microbiology, University of Debrecen, 4032 Debrecen, Hungary; gecela@post.sk

The conversion of grape juice into wine is a complex biochemical process involving alcoholic fermentation, production of wide range of metabolites and interactions of yeast strains, bacteria and fungi. During propagation, yeasts utilise sugars and other components of grape juice as substrates for their growth, converting these to ethanol, carbon dioxide and other metabolites that contribute to the chemical composition and sensory properties of the wine. In the more than a century and a half since Pasteur’s pioneering research, much has been revealed about the role of yeasts in the production of wine. Investigations carried out in wine-growing regions across the world identified over one hundred species in mature grape, grape juice, fermenting must/wine and aging wine (e.g., chapters of the book, *Yeasts in the Production of Wine* [1]). The contribution of the species to the process of vinification is diverse. The oxidative (mostly basidiomycetous) yeasts colonising the ripening grape or propagating on the surface of the aging wine (e.g., [2,3,4,5]) do not take part in fermentation but can affect the sensory properties of the wine with their secreted metabolites and cellular components released during cell lysis. Being dependent on the presence of oxygen, these yeasts die in the grape must. The fermenting yeast population is established by ascomycetous yeasts which either colonised the grape before harvest or originate from the winery environment. The majority of these yeasts are poor at fermentation, sensitive to the increasing alcohol concentration and hence, die off shortly after the onset of fermentation or are rapidly outcompeted by *Saccharomyces* strains, the “major” fermentative wine yeasts that produce most of the wine alcohol and are more tolerant to it (e.g., [6]). Only a few non-*Saccharomyces* strains can persist until the end of fermentation (e.g., [7,8,9]). Some of them pose a threat to the aging wines containing residual sugar by causing microbial instability such as secondary fermentation [10]. All yeasts of the fermenting populations affect the properties of the wines; some can play significant roles and others only have minor impact. The effects can be beneficial, detrimental or ambivalent (positive in some circumstances and negative in other circumstances) (e.g., [11]). In modern technologies, the positive properties of the non-*Saccharomyces* strains are exploited to refine the quality of the wine. Their cultures are inoculated into the must together with *Saccharomyces* starters or later in more advanced stages of fermentation. They can exert positive effects directly by the reduction of the concentration of certain metabolites and enriching the wine with novel metabolites not produced by *Saccharomyces* or indirectly by affecting the growth and metabolism of other yeasts. In spontaneously fermenting wines, these inter-strain interactions can modulate (reshape) the composition and diversity of the fermenting yeast populations (e.g., [5,12]). This special issue including 12 papers highlights recent advancements in certain fields of the investigation of oenological yeasts.

The review paper by Santiago Benito [13] covers the literature reporting experimental results of the combined use of two non-*Saccharomyces* yeasts of very different properties: the budding yeast *Lachancea thermotolerans* (*Kluyveromyces thermotolerans*) and the fission yeast *Schizosaccharomyces pombe*. Inoculation of the grape must with mixed cultures of these yeasts is a new technology which can be proposed for the fermentation of high-sugar musts in wine-growing areas with warm climatic conditions. The author compares the often discordant data published by various researchers and concludes that co-fermentation with these yeasts appears to be suitable to solve specific winemaking problems such as those caused by low acidity, biogenic amines, ethyl carbamate, or undesirable colour losses.

Another review [14] published by the same research group in collaboration with another Spanish team provides an overview of recent experimental results on the oenological application of *Metschnikowia* yeasts. A group of species comprising the *pulcherrima* clade occurs quite frequently on ripening grape and thus, their strains are often present in the yeast population at the beginning of fermentation. Since the taxonomic differentiation of these species is hampered by the fuzzy species boundaries [15], their strains are usually assigned to one of the species, to *M. pulcherrima* (*Candida pulcherrima*). The authors adhere to this practice in their article and consider all strains described in the oenological literature conspecific strains of *M. pulcherrima*. This approach might account for the variability of certain oenologically relevant properties of strains. From the comparison of the results published by many laboratories on many strains, the authors conclude that the main advantages of using *Metschnikowia* strains is their positive influence on wine aroma in general, the lower ethanol concentration and the higher glycerol level in the wine. However, certain strains were found to negatively affect wine quality by intense production of certain unpleasant metabolites.

*Metschnikowia* is also involved in two studies focused on the modulation of volatile contents of wine. In the work by Lee and Park [16], nine non-*Saccharomyces* yeast strains isolated from grapes and food products were studied to compare their properties with a commercial *S. cerevisiae* strain. Four of them, including a *M. pulcherrima* isolate, turned out to have β-glucosidase activity. Wines co-fermented with them and the *S. cerevisiae* strains had increased levels of linalool, citronellol and geraniol compared to the wine fermented only with the *S. cerevisiae* strain. The Zhang et al. [17] paper describes the effect of extracted β-glucosidases of three non-*Saccharomyces* strains on flavour complexity of wines. The idea behind these experiments is that many flavour components can become volatile when their glycosidic bonds to other compounds are hydrolysed. The goal could also be achieved by inoculating these yeasts into the fermenting must, but then their interactions with *Saccharomyces* could have adverse effects on the fermentation process. The presented results show that the supplementation of the must with these enzymes has no negative effect on the fermentation process and the major characteristics of the wines but increases the content of terpenes, esters and fatty acids.

Nitrogen availability is a key parameter for grape must fermentation because nitrogen is essential for yeast growth and metabolic activity (e.g., [12]). Deficiency of nitrogen sources in the grape must causes stuck or sluggish fermentation. Amino acids are the main source of nitrogen in the grape must although aromatic amino acids are difficult to use by *Saccharomyces*. The paper by Álvarez-Fernández et al. [18] compares the kinetics of aromatic amino acid Catabolism and the production of 33 metabolites/derivatives in wines inoculated with *S. cerevisiae*, and sequentially with *S. cerevisiae* and *Torulopsis glabrata*. The results demonstrate that the presence of *T. glabrata* can significantly modulate the catabolic processes and the proportions of tryptophan and tyrosine derivatives. Kessi-Pérez et al. [19] approach the problem of utilisation of nitrogen sources in a different way. Based on numerous previous observations demonstrating that nitrogen consumption and utilisation, as many other traits of oenological interest, are polygenic, they developed a breeding programme. Previous studies indicated that the different efficiency of *Saccharomyces* strains in nitrogen source utilisation is due to different allele combinations of the involved genes. The authors assumed that a better efficiency can be achieved through the combination of favourable alleles of different strains by crossing them and selecting recombinant descendants/segregants of the hybrids. With this in mind, they isolated strains from winemaking environments and hybridised them. One of the hybrid derivatives was tested for wine production from grape must unsupplemented with nitrogen on a pilot scale and was found to be as efficient at fermentation as the control commercial strain.

The paper published by Mandakovic et al. [9] addressed the diversity of fungal communities of grape must at the start and at the completion of fermentation. The ITS-based metabarcoding analysis identified sequences forming 188 OTUs. However, a large proportion of the OTUS could not be assigned to species and many OTUs were assigned to organisms whose DNA can be considered a specific environmental pollution in the samples. The analysis did not take into consideration the intraspecies and intragenomic diversity of ITS sequences, and thus, the real diversity could be much lower than that suggested by the high number of OTUs. In order to select indigenous yeasts to use as potential new starters, a few yeasts were isolated from unfermented grape must and tested for certain oenological properties. Although the isolated non-*Saccharomyces* strains showed high fermentation rates and proved superior to the *Saccharomyces* control strain in certain characteristics, they fell behind in the competition experiments.

The study of Deed and Pilkington [20] reports on the results of the comparison of the ability of genetically diverse *S. cerevisiae* strains isolated from different environments and geographical locations to ferment grape juice. The results demonstrate that geographical origin plays a lesser role in the determination of the strain‘s fermentative ability than lifestyle (the environment from which the strain originated) or the genetic lineage (domestication lineage).

During the fermentation of the grape juice, the microorganisms are exposed to continuously changing stressful conditions, such as the initial high sugar concentration, low pH, shortage of oxygen, the rapidly increasing ethanol concentration and limited nutrients. In this environment, the ability to tolerate various stresses is essential for the survival and proper activities of the yeast cells. The paper by Garrigós et al. [21] describes the consequences of the inactivation of *TSA1*, a gene coding for peroxiredoxin, a component of the antioxidant stress response. Interestingly, *TSA1* deletion did not affect grape juice fermentation in spite of reducing the fermentative capacity. It also reduced the production of acetaldehyde and acetic acid, two metabolites with strong organoleptic impact. However, further research has to be done to clarify the connections between stress response and the production of these and other metabolites which are also affected by the absence of *TSA1* activity.

The ability to cope with stress conditions characteristic of the wine fermentation process is a requirement of the applicability of non-*Saccharomyces* strains as well. *Torulaspora delbrueckii* strains have several properties that can be exploited to improve certain wine parameters, but their cells are usually less tolerant than those of *S. cerevisiae* to stresses (e.g., [22]). The paper of Velázquez et al. [23] demonstrates that the stress tolerance of *T. delbrueckii* strains can be gradually improved by multi-step (successive) selection of mutants because their tolerance increases at each step.

The paper entitled “Biological processes highlighted in *Saccharomyces cerevisiae* during the sparkling wines elaboration” [24] presents lists of proteins detected by proteomic analysis of samples of cells during the second fermentation of sparkling wines under CO_2_ overpressure and without it. Although the proteins detected are involved in general biological processes, the results can serve as a basis for further, more specific studies.

In all papers of the Special Issue, *Saccharomyces* is considered the “major” wine yeast, whereas the other species are regarded as minor players that can only modulate the fermentation process and/or certain properties of the wine. The only exception is the Csoma et al. [5] work that reports on “*Saccharomyces*-free” spontaneous fermentation. The molecular taxonomic examination of high numbers of yeasts isolated from fermenting and ageing high-sugar botrytized wines identified 13 non-*Saccharomyces* species, mostly known as osmotolerant spoilage yeasts or biofilm-producing yeasts. None of the wines had homogeneous yeast populations. Antagonistic and synergistic interactions of the species and the genetic segregations of certain clones shaped the yeast biota.

Overall, this Special Issue, which includes the papers of scientists of 11 research teams around the world, presents valuable new data and ideas on wine yeasts and their application to technological innovations and wine quality improvement. I would like to thank all the authors for submitting their excellent works, the reviewers for their valuable time and important comments, and the members of the *Microorganisms* Editorial Office for continuous support.
